# Understanding variability in the benefits of N_2_-fixation in soybean-maize rotations on smallholder farmers’ fields in Malawi

**DOI:** 10.1016/j.agee.2017.05.008

**Published:** 2018-07-01

**Authors:** D. van Vugt, A.C. Franke, K.E. Giller

**Affiliations:** a Plant Production Systems, Wageningen University, PO Box 430, 6700 AT Wageningen, The Netherlands; b International Institute of Tropical Agriculture, Chitedze Research Station, PO Box 30258, Lilongwe 3, Malawi; c Soil, Crop and Climate Sciences, University of the Free State, PO Box 339, Bloemfontein 9300, South Africa

**Keywords:** Natural abundance method, Crop rotation, Nitrogen fixation, Soil fertility, Yield variability

## Abstract

Soybean production can contribute to the nitrogen economy of smallholder farming systems, but our understanding of factors explaining variability in nitrogen fixation and rotational benefits across farms and regions is limited. Biological nitrogen fixation (BNF) was quantified with the natural abundance method in 150 farmer-managed soybean plots under different varieties and inputs in Dowa, Mchinji and Salima districts of Malawi. Soybean yielded on average 1.2 t ha^−1^ grain and the above-ground biomass at mid pod filling (R5.5) was 2.8 t ha^−1^ and contained in total 63 kg ha^−1^ nitrogen derived from the atmosphere (Ndfa). Locally sourced varieties obtained a larger %Ndfa (65%) than the ‘improved’ variety Nasoko (53%). The %Ndfa was positively associated with soil sand content, sowing date, plant population and biomass accumulation, but it was not affected by inoculation with rhizobia or the combination of inoculation and NPK fertiliser application. Quantities of N_2_ fixed differed between regions and years, and was enhanced by applying inoculant and fertiliser together, leading to more biomass accumulation and larger grain yields. Soil available P and exchangeable K contents also increased the total amount of N_2_ fixed. In a related trial, continuous maize yields were compared with maize following soybean in 53 farmer-managed fields. Average yield in continuous maize was 2.5 t ha^−1^, while maize after soybean produced 3.5 t ha^−1^ (139% of continuous maize). Farmers with higher maize yields, who applied external nutrient inputs, and with a larger value of household assets achieved greater yield responses to rotation with soybean. A relative yield increase of more than 10% was observed on 59, 90 and 77% of the fields in Dowa, Mchinji and Salima respectively. We conclude that fields of soybean and maize that receive adequate nutrient inputs and good management to ensure good yields benefit most in terms of quantities of N_2_ fixed by the legume and the yield response of the following maize crop. The results suggest that the promotion of soybean-maize rotations should be done through an integrated approach including the promotion of appropriate soil and crop management techniques. Furthermore, they suggest that wealthier households are more likely to apply adequate nutrient inputs and good crop management practices and are likely to receive larger maize yield responses to the incorporation of soybean.

## Highlights

• N_2_-fixation depends on genetic, environmental, management and socio-economic factors.
• More productive and wealthier farmers benefit most from soybean-maize crop rotation.
• Soybean-maize rotations should be promoted with integrated soil and crop management.

## 1 Introduction

In Southern Africa, maize is the most dominant crop and is produced on 47% of cultivated land (FAO, 2014). It is the main crop for smallholder farmers who constitute the majority of the rural population and depend mainly on rain fed agriculture for food and income generation. In 2011 the average smallholder landholding size in Malawi was 0.8 ha and over 80% of this land was cultivated with maize (IFAD, 2011). Fertiliser use is highly variable among African smallholder farmers, but generally resource constrained farmers apply few external inputs, which leads to poor yields and nutrient depletion (Waddington et al., 2004, Vanlauwe and Giller, 2006). As a result nitrogen is widely limiting and farmers find themselves in a poverty trap where increasing nutrient and organic matter depletion may eventually result in non-responsive degraded soils (Tittonell and Giller, 2013). Increasing the share of legumes can contribute to sustainable intensification of maize-based cropping systems by enhancing the input of abundantly available atmospheric N_2_ through biological nitrogen fixation (BNF) (Mhango et al., 2013). Legumes fix on average 30–40 kg of N_2_ for every ton of shoot dry matter produced and can contribute to improved soil fertility and enhanced yields of a subsequent cereal crop (Peoples et al., 2009). Crop diversification with legumes can better meet caloric and protein needs of farm households if farmers adopt species that perform well under variable rainfall patterns (Snapp et al., 2014). Legumes also provide nutritional benefits through the addition of proteins to starch-based diets (Bezner Kerr et al., 2007). There is scope for enhancing productivity of edible and marketable grain legumes (Mhango et al., 2013) such as soybean (*Glycine max* (L.) Merril.) for the expanding market in Southern Africa for livestock feeds, edible oils and human foods (Tichagwa and Rusike, 2009). Soybean fixes on average approximately 50–60% of its nitrogen (Hardarson and Atkins, 2003, Salvagiotti et al., 2008) though ranges of 9 to 91% have been reported (Franke et al., 2017).

Challenges to increase the area under legumes in southern Africa include high labour costs associated with legume cultivation, poor yields (Waddington and Karigwindi, 2001, Franke et al., 2014) and poor access to quality seed, inputs and output markets (Snapp et al., 2002, Mtambanengwe and Mapfumo, 2009). Farmers usually prioritise maize above legumes as maize yields and returns to labour are often better. However, including soybean into the cropping system can become attractive when the rotational benefits to maize in terms of yield, food security and profitability are considered (Franke et al., 2014). This is especially the case if good productivity of the legume can be assured through good management practices such as the application of inoculants, inorganic fertiliser or compost manure (Hati et al., 2006, Ndakidemi et al., 2006, Van Vugt et al., 2016). The amounts of nitrogen fixed may vary across different agro-ecological zones (Ojiem et al., 2007). On poor degraded soils, manure application can enhance nitrogen fixation (Zingore et al., 2008). The percentage of nitrogen derived from the atmosphere (%Ndfa) can be reduced by nitrogen fertiliser application (Hardarson and Zapata, 1984, Salvagiotti et al., 2008) and soil water deficits (Sinclair et al., 2007). Self-nodulating promiscuous types of indeterminate soybean can fix more nitrogen than high harvest index grain type varieties (Snapp et al., 1998) due to their longer growing period and better ability to nodulate with indigenous *Bradyrhizobium* strains in the soil (Mpepereki et al., 2000). Therefore, when seeds are not inoculated, promiscuous soybean varieties tend to confer a larger residual benefit on the following maize crop than specific varieties (Kasasa et al., 1999). However, farmers often prefer shorter duration grain-type varieties as they give quicker returns on investments (Snapp and Silim, 2002; Adjei-Nsiah et al., 2008).

Nitrogen fixation, the yield performance of legumes and the residual benefits to a following cereal crop depend on a range of environmental and crop management factors which in turn are a reflection of farmers’ socio-economic conditions. Smallholder farming systems are very heterogeneous in biophysical and socio-economical characteristics (Ojiem et al., 2006) and agronomic research is often not adapted to include this variability when identifying options to enhance productivity (Vanlauwe et al., 2016). While legumes are well known to fix N_2_ and improve yields of subsequent cereals in SSA, a high variability across smallholder farmers in socio-economic and biophysical conditions implies these benefits are also highly variable. We are unaware of studies in SSA that quantify and attempt to explain the variability in BNF by soybean and maize yield response to crop rotation across a wide range of smallholder farmers’ fields. This is however crucial for improved tailoring of legume-based technologies to those farmers where impact is likely to be largest. Therefore, this study aims to quantify and understand the variability and factors behind BNF and rotational effects of including soybean in maize-based rotations, based on a large number of farmer-managed trials in Central Malawi.

## 2 Materials and methods

### 2.1 Trial lay-out and treatments

On-farm experiments were conducted in Dowa, Mchinji and Salima districts (also referred to as regions) in Central Malawi in the 2009/10 and 2010/11 growing seasons, in this study referred to as the 2010 and 2011 seasons respectively. Central Malawi has a uni-modal rainfall distribution with rains starting early December and continuing for four months. Long term precipitation averages are in the range of 900–950 mm per year (Hijmans et al., 2005). A nutrient management (NM) trial and a crop management (CM) trial was established with 12 treatment blocks for each trial in each region in each year giving a total of 72 blocks per trial. Farmers hosted a single replicate block of one of the trials with five non-randomly assigned treatments. In the NM trials inoculant, fertiliser and compost manure treatments were assigned to five plots of 10 by 10 m. The CM trials consisted of five plots with variety, weed management, plant population, and pest and disease control treatments. In Dowa district, trials were established around Msakambewa trading centre (13°33′S, 33°54′E) at 1200–1400 m above sea level (masl), in Mchinji around Kachamba village (13°44′S, 33°20′E) at an altitude of 1050–1150 masl and in Salima around Chitala research station (13°40′S, 34°15′E) at 550–650 masl. The major soil types in Dowa and Mchinji are Chromic luvisols and in Salima Eutric cambisols. For a more detailed description of the NM and CM trials, see Van Vugt et al. (2016).

In this study we use data collected from a subset of the treatments and farmers participating in the NM or CM trial. To assess BNF for different varieties and input levels, five blocks from the twelve replicate blocks per region per year were selected from each trial, resulting in a total of 60 blocks (5 blocks × 3 regions × 2 years × 2 type of trials; 30 blocks assessed in each trial), hosted by 56 farmers (four farmers in Mchinji hosted a trial block in both years). BNF data were collected from the following treatments:

• T1 (*n* = 30) inoculated soybean seed of unknown variety procured from local markets in each region
• T2 (*n* = 30) variety Nasoko without any inputs
• T3 (*n* = 60) variety Nasoko with inoculation
• T4 (*n* = 30) variety Nasoko with inoculation and application of 300 kg ha^−1^ compound fertiliser Super D containing 10% N, 8% P and 20% K.

Nasoko is a commonly grown, specifically-nodulating variety that, unlike more ‘promiscuous’ varieties that can effectively nodulate with a large diversity of indigenous rhizobia in the soil (Giller et al., 2011), needed to be inoculated. The applied inoculant was manufactured at the Soil Productivity Research Laboratory, Marondera, Zimbabwe and contained the *Bradyrhizobium japonicum* strain MAR 1491 (Giller et al., 2011). T1 was a treatment in the CM trials, T2 and T4 in the NM trials, and T3 in both trials. We refer to this set of treatments in which we assessed BNF as the ‘BNF trial’ and since we use a flexible linear mixed model (REML) tool for analysis we can still analyse this unbalanced design with treatments that were done at different farms. Apart from the described treatments, farmers were free to manage the trial plots according to their own preferences

To assess the residual benefits of soybean on a subsequent maize crop 53 farmers (17 in Dowa, 19 in Mchinji and 17 in Salima) participated in a crop rotation trial. These farmers all hosted a trial with a treatment plot ‘Nasoko with inoculation’ in 2010, but only 21 of these plots (7 in Dowa, 9 in Mchinji and 5 in Salima) were also part of the BNF trial. Soybean did not receive any external nutrient inputs. In 2010 farmers typically produced maize on a field near the soybean plot on a similar soil type. At the start of the 2011 season a plot of 10 by 10 m was demarcated on this field previously cultivated with maize. All farmers subsequently sowed their own maize seeds on both plots, resulting in a soybean-maize rotation (SM) and a continuous maize (MM) treatment. Farmers were instructed to plant both maize plots on the same day and apply their common crop husbandry practices and inputs. This trial is referred to as ‘rotation trial’ in this study.

### 2.2 Data collection

Daily rainfall was collected by a field technician and three farmers in each region. Composite soil samples (0–20 cm) were collected in the BNF trial by taking five subsamples from each block. Samples were mixed, air-dried, crushed and sieved through a 2-mm sieve and analysed at IITA-Malawi and Chitedze Research Station for soil organic carbon (SOC) (Walkley-Black), available P (Bray-1), soil pH (CaCl_2_), exchangeable K (Mehlich 3 method) and texture. In 2011 soil samples were collected from the two maize plots of the rotation trial and analysed for SOC, available P and soil pH following the same methods.

Farmers’ practices in the BNF trial recorded by field technicians included actual sowing and weeding dates, the number of ridges and their spacing, the number of rows sown per ridge and the number of plants counted on two selected ridges in each plot at 3 weeks after sowing (WAS). Weed pressure was scored visually from 1 (<10% of the plot surface covered with weeds) to 5 (>90% of the plot surface covered with weeds) at 5, 8 and 11 WAS. Above-ground biomass samples of soybean were collected in the two seasons in all plots in the BNF trial at R5.5 (mid pod filling) growth stage. Sub-samples from three quadrants of 0.5 × 0.5 m were combined into one composite biomass sample per plot. Broad-leaved weed species were sampled as reference plants from unfertilized un-weeded soybean plots or from border margins in case the plots were all weeded. The weed species sampled in Dowa were *Ageratum conyzoides* (11 fields) and *Leucas martinicensis* (7 fields), in Mchinji *Bidens pilosa* (all fields) and in Salima *Bidens pilosa* (13 fields), *Bothriocline laxa* (5 fields) and *Leucas martinicensis* (1 field). The 150 soybean and 60 broadleaved weed samples were oven-dried until constant weight and ground to powder with an electric mill. They were weighed at 7 mg on a microbalance, stored in tin capsules, and analysed for nitrogen content (%) and δ^15^N at the UC Davis Stable Isotope Facility using a continuous flow isotope ratio mass spectrometer. The ^15^N natural abundance method was applied to estimate nitrogen fixation (Unkovich et al., 2008). The percentage of nitrogen derived from the atmosphere (%Ndfa) was calculated using the formula *%Ndfa* *=* *((δ^15^N_ref_ − δ^15^N_fix_)/(δ^15^N_ref_ − B))* *×* *100*, where ‘ref’ are non-fixing and ‘fix’ are nitrogen fixing plants grown under the same conditions, and *B* is the δ^15^N of the N_2_-fixing plant grown with N_2_ as the sole external nitrogen source. The *B* value for soybean used was −2.00 (Boddey et al., 2000, Ojiem et al., 2007). The formula *gNdfa* *=* *N yield *× *%Ndfa/100*, was then used to calculate the amount of nitrogen fixed per ha based on the nitrogen content in the samples and the total dry biomass accumulated per ha at the time of sampling. The ^15^N natural abundance method only works if the δ^15^N of the legume falls between the ‘*B*’-value and the δ^15^N of the reference plant. Plots for which this condition was not met were excluded from further analysis. At crop maturity, plots were harvested excluding the outer ridges and the 1.5 m ridge-length from which the biomass sub-samples were collected. Harvested plants were threshed and weighed, and a sub-sample of the grain was taken from each plot to assess moisture content. Yields were adjusted to 13% grain moisture content. Biomass is presented as above-ground dry matter weight. Socio-economic characteristics including gender, age, arable land area (ha), available family labour (ME), value of assets (USD) and livestock ownership (LU) were collected through structured interviews with farmers participating in all soybean trials as explained in Van Vugt et al. (2016).

In the second season of the rotation trial (2011), structured questionnaires were conducted with all participating farmers to record input application and agronomic practices in the maize plots in the two seasons. The method of data collection in the 53 soybean plots in the first season was similar to the BNF trial, except that in the rotation trial oven-dried biomass samples were only analysed for nitrogen content (%) and δ^15^N in the 21 plots that also formed part of the BNF trial. At maize maturity in 2011, both plots were entirely harvested and maize was dried, shelled and kept in 50 kg bags at the household until the technician came to weigh the grain. Therefore, this study presents maize yields measured under storage conditions with an estimated moisture content of 12 to 15.5%.

### 2.3 Data handling and analysis

Linear mixed model (REML) analysis was used to test the effects of treatments in the BNF trials on δ^15^N, %Ndfa, total N_2_ fixed (kg ha^−1^) and grain yield (t ha^−1^), while testing for interactions between treatments, years and regions. Similar analysis was done to assess the effect of region on maize yields and the yield response to crop rotation with soybean. Since average values are not very informative due to large variability in responses across farms we presented data in cumulative frequency curves (Vanlauwe et al., 2016). The next step was to explore which factors contributed to the variability in the dependant variables %Ndfa, total N_2_ fixed, soybean yields, maize yields and yield responses. To avoid erratic model outputs due to collinearity, independent variables were associated with the dependant variables in separate analyses. REML is a flexible tool for analysis that can include unbalanced and categorical data and can be used to compensate for confounding factors and was used in similar studies to explain variability (Franke et al., 2016; Ronner et al., 2016). We included region and/or year as random factors in the model when they affected the dependant variable (p < 0.05). Continuous independent variables in the fixed model included sowing date, first weeding date, weed pressure score (1–5), plant population density, biomass accumulation, plant height, soybean grain yield, soil texture, soil OC, P, K and pH and the socioeconomic characteristics arable land area, age of farmer, available family labour, value of assets and livestock ownership. Categorical factors included gender, external nutrient input (yes/no), improved maize variety (yes/no) and crop residue management (compost, incorporation in the soil, burnt). Input levels in maize were determined through questionnaires resulting in rough estimates of quantities of urea (46% N) and/or NPS (23:21 +4S) applied per hectare. Since we could not assign reliable quantities of N and P to each field we included input level as a categorical factor (with or without inputs) in the REML. We used Spearman’s Rank Correlations test to determine if the effect of a continuous independent variable on the dependent variable was positive or negative. In the 21 sites where the BNF and rotation trials overlapped, we also tested for correlations between soybean yield components and N_2_ fixation data and the following maize yields and yield responses to rotation. All statistical analysis were done using Genstat 18th edition.

## 3 Results

### 3.1 Socio-economic and biophysical characteristics of the farmers

The 83 farmers who participated in the trials had different socio-economic and biophysical characteristics (Table 1). In Dowa a larger percentage of women hosted a trial and the households were poorer in terms of the value of assets, since field technicians in Dowa targeted vulnerable female farmers, while in the other districts a more random selection of farmers was made. In Salima participating farmers were relatively young and families had less labour available than in the other regions. Farmers in Mchinji kept more livestock. Soils in Dowa contained more OC, while in Salima soil pH was higher and more favourable for crop growth. There was a large variability in soil available P content within each region. Soil properties in soybean-maize plots were not different from the continuous maize plots in any of the three regions (data not shown). Rainfall was more than the 50-year average in both seasons except for Mchinji in 2011. In Salima in 2010 over 80% of the total rain fell in February. Daily rainfall data in the three regions during the trials are presented in Van Vugt et al. (2016).

**Table 1:** Socio-economic and biophysical characteristics of participating farmers in three regions. Data in brackets represent standard deviations from the mean.

	Dowa	Mchinji	Salima	Total/Fpr^a^/Mean
Participation in trials				Total
Only BNF^b^ trial (*n*)	13	6	11	30
Only rotation trial (*n*)	9	10	8	27
Both trials (*n*)	8	9	9	26

Socio-economic characteristic				F pr
Female participants (%)	72	31	32	
Age of farmer	51 (13)	47 (14)	32 (9)	<0.001
Arable land (ha)	1.4 (0.86)	2.9 (2.6)	3.4 (3.8)	0.017
Available family labour (ME^c^)	4.4 (2.4)	4.1 (1.8)	3.1 (1.3)	0.041
Value of assets (USD)	81 (152)	288 (334)	250 (463)	n.s.
Livestock ownership (LU^d^)	0.7 (1.8)	3.1 (5.6)	1.1 (2.4)	0.036

Soil data both trials				F pr
SOC^e^ (g kg^−1^)	15.3 (4.1)	8.3 (2.4)	8.8 (4.1)	<0.001
P (mg kg^−1^)	7.2 (9.7)	9.8 (5.8)	8.6 (13.7)	n.s.
pH (CaCl_2_)	4.8 (0.4)	4.6 (0.3)	5.4 (0.6)	<0.001

Soil data BNF trials only				F pr
K (cmol kg^−1^)	5.4 (3.5)	2.5 (1.2)	6.1 (2.2)	<0.001
Clay (g kg^−1^)	402 (80)	282 (127)	285 (121)	<0.001
Silt (g kg^−1^)	146 (36)	125 (75)	143 (73)	n.s.
Sand (g kg^−1^)	452 (89)	594 (188)	572 (183)	0.002

Climatic data				Mean
Rainfall 2009/10 (mm)	979	1257	1199	1145
Rainfall 2010/11 (mm)	1278	756	1106	1047
Rainfall 50 years average^f^	905	952	946	934

a Fpr = the probability of no difference between regions calculated through REML analysis. Fpr > 0.05 means no significant difference (n.s.) between regions.

b Biological Nitrogen Fixation.

c Men Equivalent.

d Livestock Units.

e Soil Organic Carbon.

f Source: Hijmans et al. (2005).

### 3.2 BNF trial

#### 3.2.1 Farmers’ practices and yields

The BNF trial plots were established on average 20 days after the first effective sowing rains, though differences in the onset of the rains between years and regions, and in farmers’ practices resulted in a wide range of sowing dates (Table 2). Fields in Dowa had larger plant populations compared with Mchinji and Salima. Mean soybean grain yields were 1.47 t ha^−1^ in Dowa, 1.14 t ha^−1^ in Mchinji and 0.99 t ha^−1^ in Salima. The average yields did not differ much between regions and years in Dowa and Mchinji, but yields of 0.38 t ha^−1^ in Salima in 2010 were much smaller than 1.60 t ha^−1^ in 2011 (Table 2). This resulted in a strong region by year effect on both biomass and grain yields. The poor performance in Salima 2010 can be explained by the erratic rainfall distribution that resulted in dry spells after sowing. This was exacerbated by grasshoppers that damaged emerging plants. Strong weed pressure (Table 2) also contributed to poor yields in 2010. In 2011 rainfall was more evenly distributed and weed pressure was less.

**Table 2:** Farmers’ crop management practices and soybean characteristics in the biological nitrogen fixation trial.

	*n*	Dowa	Mchinji	Salima	Mean	SED^a^
Date of sowing rains (SR)						
2010	75	15 Dec	11 Nov	21 Dec		
2011	75	5 Dec	24 Nov	2 Dec		

Sowing date (days after SR)						Y = 0.99** R = 1.21*** R x Y = 1.71
2010	75	26	22	8	19	
2011	75	28	24	13	22	

First weeding (DAP)						Y = 1,68*** R = 2.05* R x Y = 2.89**
2010	63	12	22	21	19	
2011	67	30	30	25	28	

Weed pressure (1–5)						Y = 0.09 R = 0.12*** R x Y = 0.16***
2010	73	1.9	1.4	2.4	1.9	
2011	60	2.2	1.6	1.8	1.8	

Plant population (1000 pl ha^−1^)						Y = 17.5* R = 21.4*** R x Y = 30.3**
2010	75	412	266	205	294	
2011	75	308	207	257	257	

Plant height (cm)						Y = 1.76 R = 2.15** R x Y = 3.04***
2010	75	47	53	40	47	
2011	75	57	44	48	50	

Biomass dry weight (t ha^−1^)						Y = 0.23*** R = 0.28*** R x Y = 0.40***
2010	73	2.8	2.7	1.1	2.2	
2011	75	4.6	2.3	2.9	3.3	

Grain yield (t ha^−1^)						Y = 0.11*** R = 0.13** R x Y = 0.19***
2010	75	1.4	1.1	0.4	1.0	
2011	75	1.6	1.2	1.6	1.5	

a SED = Standard error of difference between means. Y = Year, R = Region, * p < 0.05, ** p < 0.01, *** p < 0.001.

#### 3.2.2 ^15^N natural abundance signatures in soybean and reference plants

The average δ^15^N values in the BNF trials were +0.80‰ for soybean shoots and +3.85‰ for broad leaved weeds species (Table 3). The δ^15^N of the local soybean varieties (−0.12‰) was smaller (p < 0.01) than for Nasoko (+0.91‰) across the three sites. The soybean δ^15^N was not affected by region or year but the δ^15^N of broad-leaved weed species were smaller in Salima. A combination of inoculant and fertiliser application resulted in smaller δ^15^N values compared with plots that received no inputs.

**Table 3:** Shoot δ^15^N (‰) of soybeans and weed reference plants in the biological nitrogen fixation trial in three regions in central Malawi. Data in brackets represent standard deviations from the mean.

Trial/Treatment		Shoot δ^15^N (‰)			
	*n*	Dowa	Mchinji	Salima	Mean
Soybean shoots					
Local variety inoculated	30	0.54 (0.83)	−0.01 (1.06)	−0.90 (0.60)	−0.12 (1.02)
Nasoko no inputs	30	1.23 (2.19)	1.41 (2.06)	2.19 (1.34)	1.61 (1.88)
Nasoko inoculated	60	1.11 (1.35)	0.73 (1.26)	0.89 (1.66)	0.91 (1.42)
Nasoko inoculated with fertiliser	30	0.95 (1.40)	0.25 (1.53)	0.94 (0.85)	0.71 (1.29)
Total/Mean soybean shoots	150	0.99 (1.46)	0.62 (1.51)	0.80 (1.61)	0.80 (1.52)

Broad leaved weed reference plants	60	4.13 (1.22)	4.51 (1.72)	2.92 (1.19)	3.85 (1.54)
SED^a^_(SoybeanTreatment)_	0.35**				
SED_(BroadleavedweedsRegion)_	0.28***				

a SED = Standard error of difference between means. ** p < 0.01, *** p < 0.001.

#### 3.2.3 Variability in%Ndfa, total N_2_ fixed and grain yields

**Fig. 1:**
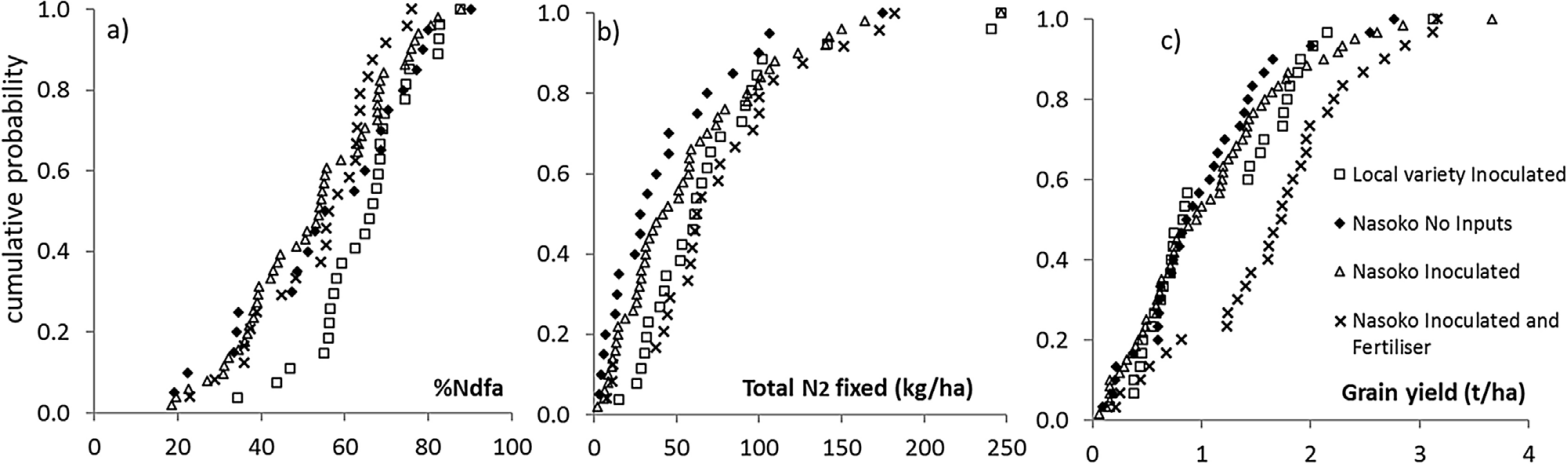
Cumulative probability charts of a) Percentage of Nitrogen derived from the atmosphere (%Ndfa) by soybean, b) total quantity of N_2_ fixed and c) soybean grain yields.

The average%Ndfa of soybean was 57% in Dowa, 58% in Mchinji and 54% in Salima (n.s.) and did not differ between the years. The local varieties fixed a larger percentage of N_2_, while inoculation and fertiliser treatments did not affect%Ndfa (Table 4). There was a large variability in%Ndfa across farms, also within treatments (Fig. 1a). Several factors contributing to this variability were identified in the REML analysis (Table 5). Plant population and biomass accumulation were positively associated with%Ndfa. Delayed sowing also correlated with a larger%Ndfa. Clay content correlated negatively and sand positively with%Ndfa. Soybean plots hosted by male farmers (*n* = 93) fixed 61% N compared to 50% on female farmers’ fields (*n* = 57). Male farmers’ fields contained more sand (580 g kg^−1^ versus 470 g kg^−1^; p = 0.01) and less clay (290 g kg^−1^ versus 390 g kg^−1^; p < 0.001) than female farmers’ fields. The percentage of female farmers that participated in the BNF trial varied by region (57% in Dowa, 20% in Mchinji and 33% in Salima) and soils in Dowa contained more clay than in Mchinji and Salima (Table 1). Despite this, there was no interaction between the variables gender and region (p = 0.15), soil texture and region (p = 0.8), or gender and soil texture (p = 0.07) in the effect on%Ndfa.

**Table 4:** %Ndfa, total N_2_ fixed, biomass yields and grain yields for different soybean varieties and input levels and in different regions. Data in brackets represent standard deviations from the mean.

Treatment	*n*	Ndfa^b^ (%)	Total N_2_ fixed (kg ha^−1^)	Biomass yield^c^ (t ha^−1^)	Grain yield (t ha^−1^)
Local variety inoculated	27	65.0 (12.4)	75.7 (57.3)	3.10 (1.88)	1.14 (0.71)
Nasoko no inputs	20	56.7 (20.2)	45.0 (43.8)	2.27 (1.54)	1.02 (0.65)
Nasoko inoculated (I)	51	53.0 (17.6)	57.8 (50.0)	2.48 (1.71)	1.08 (0.75)
Nasoko, I and fertiliser	24	54.3 (14.5)	76.7 (46.9)	3.48 (1.54)	1.68 (0.79)
SED^a^ Treatment		4.47*	12.01*	0.41*	0.18**

Region					
Dowa	45	57.1 (16.9)	88.9 (60.9)	3.70 (1.96)	1.47 (0.73)
Mchinji	41	57.7 (14.3)	49.9 (36.7)	2.52 (1.42)	1.14 (0.61)
Salima	36	54.3 (19.9)	47.1 (37.9)	2.04 (1.27)	0.99 (0.86)
SED Region		3.79	10.71***	0.32***	0.15**
Total/Mean	122	56.5 (17.0)	63.3 (50.9)	2.76 (1.72)	1.20 (0.76)

a SED = Standard error of difference between means, * p < 0.05, ** p < 0.01, *** p < 0.001.

b Nitrogen derived from the atmosphere.

c Above-ground biomass dry weight at R5.5 growth stage.

**Table 5:** Factors affecting%Ndfa, quantities of N_2_ fixed and soybean grain yield.

Dependent variable	Type of relation^a^	*n*	Fpr	Random Factors^b^
Explanatory variables				
%Ndfa^c^				
Technology treatment	C	122	0.023	−
Clay	−	120	0.043	T
Sand	+	120	0.039	T
Sowing date	+	122	0.042	T
Plant population	+	150	0.029	−
Biomass yield	+	148	0.049	−
Gender	C	150	<0.001	T
Value of assets	−	150	0.050	T

Total N_2_ fixed				
Region	C	120	<0.001	−
Year	C	120	0.003	−
Technology treatment	C	120	0.043	R, Y
Available P	+	141	<0.001	R, Y, T
Exchangeable K	+	111	0.015	R, Y, T
Grain yield	+	150	<0.001	R, Y
Biomass yield	+	148	<0.001	R, Y
Plant height	+	148	0.002	R, Y

Soybean grain yield				
Region	C	150	0.005	−
Year	C	150	<0.001	−
Technology treatment	C	150	<0.001	R, Y
Plant height	+	148	<0.001	R, Y
Biomass yield	+	148	<0.001	R, Y
Plant population density	+	150	0.013	R, Y
Weed score	−	133	0.022	R, Y
Total N_2_ fixed	+	120	<0.001	R, Y
Net N benefit from BNF	−	120	<0.001	R, Y
Gender	C	150	0.002	R, Y, T
Value of assets	+	150	<0.001	R, Y, T

a For continuous variables ‘+’ indicates a positive and ‘−’ a negative correlation with the dependent variable; Categorical factors are indicated with “C”.

b Random factors included in the REML model: R = Region, Y = Year, T = Technology treatment.

c Percentage of nitrogen derived from the atmosphere.

The average total N_2_ fixed was 63 kg ha^−1^ and there was an effect of region (Table 4) and year with for instance 21 kg ha^−1^ fixed in Salima in 2010 and 107 kg ha^−1^ in Dowa in 2011. Like the %Ndfa, the local varieties also fixed larger quantities of N_2_ per ha, though this did not result in better grain yields (Table 4). Total N_2_ fixed increased with the combined application of inoculant and NPK fertiliser compared to the no input treatment (Table 4), though a considerable variability existed within all treatments (Fig. 1b). Total N_2_ fixed was strongly associated with plant growth traits such as grain yield, biomass yield and plant height and was positively affected by soil available P and exchangeable K (Table 5). Plants on soils containing more available P accumulated more biomass (r = 0.32, p < 0.001) and had taller plants (r = 0.19, p = 0.05).

Soybean grain yields were affected by year, region and input level (Table 2, Table 4). Average grain yields without inputs were 1.02 t ha^−1^, with only inoculation 1.08 t ha^−1^ and with inoculation plus fertiliser 1.68 t ha^−1^. The combined application of fertiliser and inoculant enhanced both biomass and grain yields compared with application of only inoculant (Table 4). The REML analysis identified additional factors that may have contributed to the large variability in yields across farms (Fig. 1c). Fields with larger plant populations were associated with better yields, while high weed pressure was associated with lower yields. Male farmers and farmers with more assets tended to have better yields (Table 5). Female farmers’ soybean grain yields were only 0.99 t ha^−1^ compared with 1.33 t ha^−1^ achieved by male farmers. Besides soil texture, we did not find any associations between other biophysical, crop management, or socio-economic variables and gender.

### 3.3 Rotation trial

#### 3.3.1 Farmers’ practices and maize yields

Most soybean plots cultivated in 2010 were preceded by cereal crops in 2009 whereas the maize plots were preceded by cereals (53%), legumes (29%) or other cash crops (Table 6). The soybean plots in Dowa accumulated most biomass and attained the largest yields followed by Mchinji and Salima. In the maize plots in 2010 most farmers in Mchinji applied a combination of ‘NPS’ (23:21 + 4S) and urea fertilisers at a rate of at 85 kg N, 11 kg P and 5 kg S ha^−1^, but in Dowa unfertilised maize was most common. The use of animal manure was rare and only three farmers used chemicals for weed, pest or disease control. At the end of the season, maize residues were mainly incorporated into the soil or burnt. Soybean residues were taken to the homestead for threshing and residues were commonly used to make compost manure, but in Salima farmers burnt the residues or took them back to the field to incorporate them into the soil.

**Table 6:** History and characteristics of plots used in the rotation trial.

Plot history before rotation trial (2009)				
Crop^a^ before soybean (% of plots)	Dowa (*n* = 16)	Mchinji (*n* = 19)	Salima (*n* = 17)	Mean
Cereals	81	84	71	79
Legumes	0	5	0	2
Other cash crops	6	0	24	10
Fallow	13	11	6	10

Crop before maize (% of plots)	Dowa (*n* = 6)	Mchinji (*n* = 16)	Salima (*n* = 16)	Mean
Cereals	83	25	50	53
Legumes	17	44	25	29
Other cash crops	0	31	25	19
Fallow	0	0	0	0

Plot characteristics trial season 1 (2010)				
External inputs in maize plots^b^	Dowa (*n* = 11)	Mchinji (*n* = 18)	Salima (*n* = 17)	Mean
NPS and Urea (%)	27	83	47	57
Animal manure (%)	9	6	12	9
External inputs in soybean plots	0	0	0	0

	Dowa (*n* = 15)	Mchinji (*n* = 17)	Salima (*n* = 15)	Mean
Yields in soybean plots (t ha^−1^)	1.13	0.76	0.33	0.74
Soybean dry biomass at R5.5 (t ha^−1^)	2.49	1.94	1.00	1.83

Use of maize residues after harvest	Dowa (*n* = 8)	Mchinji (*n* = 18)	Salima (*n* = 17)	Mean
Make compost manure (%)	13	6	6	7
Incorporate into the soil (%)	50	67	53	58
Burn (%)	38	28	41	35

Use of soybean residues after harvest	Dowa (*n* = 11)	Mchinji (*n* = 17)	Salima (*n* = 17)	Mean
Make compost manure (%)	91	94	6	60
Incorporate into the soil (%)	0	0	53	20
Burn (%)	9	6	41	20

a Cereals are maize (53) and in Salima sorghum (5); Legumes include groundnuts (8) and soybean (6); Cash crops include in Mchinji tobacco (5), in Salima cotton (8) and in Dowa sweet potatoes (1).

b Percentage of farmers applying these inputs. NPS (23:21 + 4S) and urea (46:0:0) were commonly applied at 125 kg ha^−1^ each.

Most maize plots in the second season of the rotation trial were sown in December, though in Mchinji and Salima sowing was spread out over two months (Table 7). In Dowa only 36% of the plots received external nutrient inputs, compared to 89% in Mchinji and 65% in Salima. There was much variation in the date of first weeding ranging from 14 to 70 days after sowing. Improved varieties were used by 61% of the farmers whereas the rest of the farmers cultivated local varieties. There was large variability in number of sowing stations per hectare and number of seeds per station. The average sowing rate was 57,700 seeds ha^−1^.

**Table 7:** Maize management in the second year (2011) of the rotation trial.

External inputs in trial plots (% of trials)	Dowa (*n* = 11)	Mchinji (*n* = 18)	Salima (*n* = 17)	Mean
NPS^a^, Urea and Manure	0.0	16.7	5.9	7.5
NPS and Urea	36.4	61.1	41.2	46.2
Urea or CAN^b^ only	0.0	11.1	11.8	7.6
NPS only	0.0	0.0	5.9	2.0
No inputs	63.6	11.1	35.3	36.7

Other crop management practices	Dowa (*n* = 8)	Mchinji (*n* = 18)	Salima (*n* = 17)	Mean
Sowing date				
First	−	27 Nov	11 Nov	
Median	−	12 Dec	6 Dec	
Last	−	28 Jan	15 Jan	
First weeding date (DAP)	−	30 (16–54)^c^	22 (14–70)	
Second weeding date (DAP)	−	58 (43–75)	40 (27–58)	
Improved variety^d^ (% of farmers)	50	68	65	61
Row spacing (cm)	75	84 (75–90)	77 (75–90)	79
Plant spacing (%)				
20–25 cm; 1 seed per station	25	5	29	20
40–50 cm; 2–3 seeds per station	50	95	6	50
60–90 cm; 3–4 seeds per station	25	0	65	30
Sowing rate (1000 seeds ha^−1^)	60.4	57.2	56.8	57.7

a NPS (23:21 + 2S).

b CAN is Calcium ammonium nitrate (27% N, 8% Ca).

c Data in brackets are minimum and maximum observes values.

d Includes hybrid and open pollinated maize varieties.

#### 3.3.2 Maize yields, yield responses and yield variability in the rotation trials

Mean maize grain yield in 2011 was 3.98 t ha^−1^ and yields varied between regions (Table 8) with 1.63 t ha^−1^ in Dowa, 2.94 t ha^−1^ in Mchinji and 4.37 t ha^−1^ in Salima (p < 0.001). Maize yields achieved by farmers were highly variable between and within regions with 90% of the fields having yields in the range of 0.9 to 3.4 t ha^−1^ in Dowa, 1.5 to 5.3 t ha^−1^ in Mchinji and 1.8 to 7.5 t ha^−1^ in Salima. The REML analysis identified the region and the previous crop (maize or soybean) as factors affecting maize yields (Table 9). Farmers that cultivated improved maize varieties also benefitted from better yields in plots that were proceeded by soybean, and input application enhanced maize yields in both treatments (Table 8). We did not find a relationship between soil characteristics, sowing date, sowing rate or socio-economic characteristics of the households and maize yields.

**Table 8:** Effect of region, maize variety and input application on maize yields and yield response to rotation. Data in brackets represent standard deviations from the mean.

	*n*	M-M^a^ plot (t ha^−1^)	S-M^b^ plot (t ha^−1^)	Yield response (t ha^−1^)
Region				
Dowa	17	1.47 (0.59)	1.79 (1.12)	0.32 (0.92)
Mchinji	19	2.30 (1.37)	3.59 (1.56)	1.29 (0.87)
Salima	17	3.75 (1.75)	4.98 (2.29)	1.23 (1.09)
SED^c^		0.45***	0.58***	0.32**

Variety class				
Improved^d^	27	2.98 (1.65)	4.01 (2.14)	1.23 (1.06)
Local	18	2.23 (1.57)	2.88 (1.87)	0.69 (0.97)
SED		n.s.	0.80*	n.s.

Input class				
No inputs	15	1.66 (0.93)	2.19 (1.24)	0.49 (0.96)
With inputs^e^	32	3.06 (1.72)	4.28 (2.10)	1.28 (1.00)
SED		0.60**	0.55***	0.32*

a M-M = maize after maize.

b S-M = maize after soybean.

c SED = Standard error of difference between means. For variety and input class ‘Region’ was added as a random factor in the REML, n.s. = not significant, * p < 0.05, ** p < 0.01, *** p < 0.001.

d Hybrid or open pollinated varieties.

e NPS (23:21 + 4S), urea, calcium ammonium nitrate (CAN) and/or manure.

**Table 9:** Factors affecting maize yields and absolute and relative response of maize yield to crop rotation.

Dependent variable	Type of relation^a^	*n*	Fpr	Random Factors^b^
Explanatory variables				
Maize yield (t ha^−1^)				
Region	C	106	<0.001	−
Treatment (soya or maize in 2010)	C	106	0.002	R
Variety (local or improved)	C	70	0.02	R,T
Input application (yes or no)	C	92	<0.001	R,T

Absolute response to rotation (t ha^−1^)				
Region	C	53	0.007	−
Inputs applied to maize plots (yes or no)	C	47	0.018	R
Mean site maize yield	+	53	<0.001	R
Value of assets (USD)	+	53	0.029	R

Relative response to rotation (%)				
Region	C	53	0.03	
Maize yield in control plot	−	53	0.019	R

a For continuous variables ‘+’ indicates a positive and ‘−’ a negative correlation with the dependent variable; Categorical factors are indicated with “C”.

b Random factors included in the REML model: R = Region, Y = Year, T = Technology treatment.

**Fig. 2:**
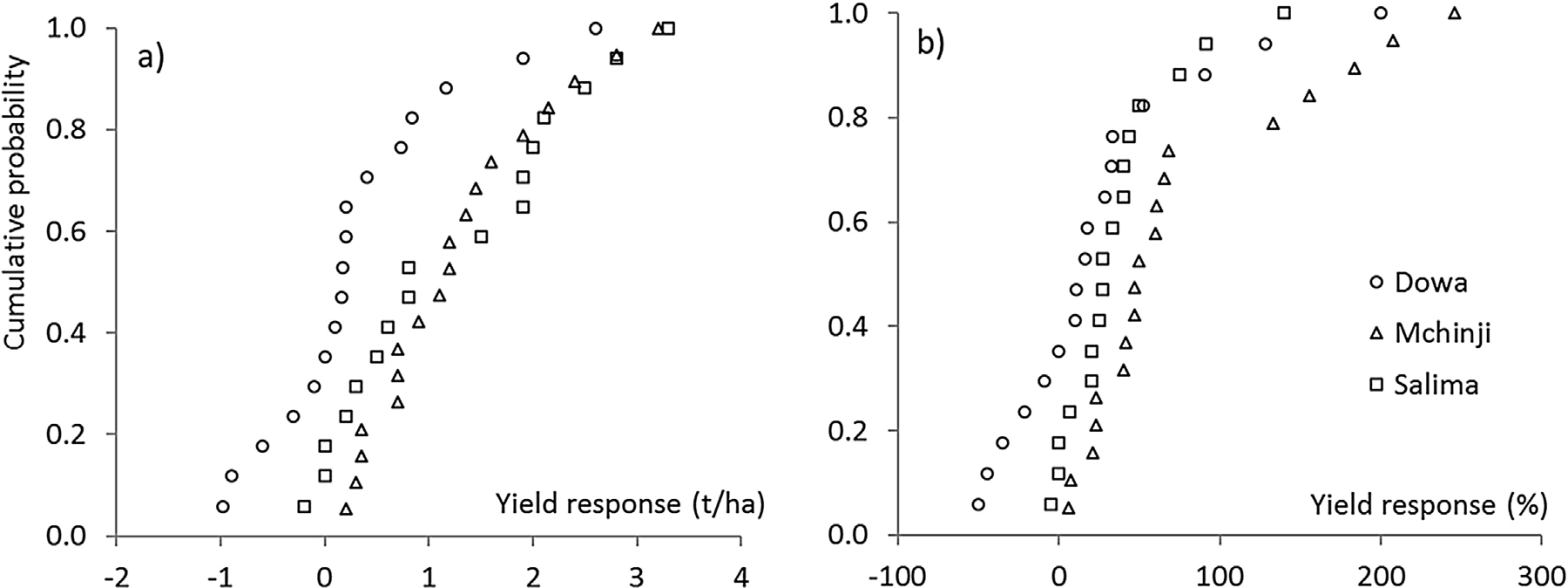
Cumulative probability of the absolute (a) and relative (b) maize yield response to crop rotation following soybean instead of continuous maize production in three regions in central Malawi.

On most farms, maize following soybean outperformed continuous maize (Fig. 2). The average yield increase of maize after soybean relative to continuous maize was 0.32 t ha^−1^ in Dowa, 1.29 t ha^−1^ in Mchinji and 1.23 t ha^−1^ in Salima (Table 8). The maize yield response was highly variable (Fig. 2a) with an overall probability of a positive response of 85%, and a 40% probability of a response above 1.0 t ha^−1^. There was variation across regions with 60, 100 and 85% of fields showing a positive response in Dowa, Mchinji and Salima respectively. Farmers that applied nutrient inputs (*n* = 32) had mean yield responses to rotation of 1.32 t ha^−1^ compared with 0.47 t ha^−1^ without input application (*n* = 15). The average site yield (average yield of the two maize plots) was strongly correlated with the absolute yield response (r = 0.52, p < 0.001) indicating that more productive farmers benefited from larger absolute yield increases. The value of assets at the household was also associated with larger yield responses (r = 0.37, p = 0.006).

Soybean as a previous crop increased maize yield on average by 39%. This relative response was affected by region with average yield increases of 22, 56 and 33% in Dowa, Mchinji and Salima respectively (Table 7). An increase of more than 10% (considered a minimum increase to be noticeable by farmers) was observed on 59, 90 and 77% of fields in Dowa, Mchinji and Salima respectively. A yield response of more than 100% was observed on 15% of the fields (Fig. 2b). Unlike the absolute yield response, the relative yield response was less in fields with a larger continuous maize yield (Table 9). There was no correlation between soybean grain yield, biomass, %Ndfa or total N_2_ fixed and the following maize yield or yield response to rotation in the 21 fields where both BNF and rotation data were collected.

## 4 Discussion

### 4.1 Methodological considerations

The farmers and fields included in the estimation of N_2_-fixation and rotational benefits represented a wide range of environmental and socio-economic conditions and crop management practices in Central Malawi. This offered a valuable opportunity to quantify and analyse the large variability in maize and soybean yields, N_2_-fixation parameters and residual effects of soybean. This type of experimentation, often conducted as part of an agricultural dissemination programme with goals other than scientific research, can easily lead to challenges with unbalanced treatment designs and confounded co-variables, which reduces statistical power and the ability to explain variability. Moreover, multiple interacting constraints typically affect crop productivity which also complicates the analyses (Fermont et al., 2009, Ronner et al., 2016). Therefore, we identified those factors that are associated with the dependant variables, but did not attempt to quantify the relative importance of each variable in explaining the overall variability. Some potentially relevant factors that could contribute to explaining variability such as daily rainfall at field level, pest and disease incidence and severity, livestock damage, and crop theft were not captured.

### 4.2 Factors affecting N_2_-fixation and soybean and maize yields

Our results show that a combination of genetic, environmental, management (GxExM) and socio-economic variables affect N_2_-fixation and soybean and maize yields (Tables 5, 8 and 9). Locally procured, undefined varieties had a larger%Ndfa (65%) than variety Nasoko (53%) receiving inoculation, while observed values were within the range reported in literature (Salvagiotti et al., 2008). The%Ndfa was not affected by region and year (Table 5). While research in Kenya showed that differences between agro-ecological zones in terms of soil fertility and rainfall can affect the %Ndfa (Ojiem et al., 2007), in our study the regions may not have been sufficiently distinct to affect %Ndfa. However, within regions a larger percentage of N_2_ was fixed on soils with a relatively high sand content (Table 5). A possible explanation for this could be that clay soils can store more organic N suppressing N_2_ fixation (Schipanski et al., 2010) (Giller et al., 1997). The%Ndfa was not affected by inoculation or fertiliser application (Table 4 and Fig. 1a). Van Vugt et al. (2016) found that the same inoculant applied in a larger number of farmers’ fields (*n* = 63) did not enhance grain yields, which could indicate that the inoculant was not very effective. Reported yield responses to inoculant application on smallholder farmers’ fields are highly variable and there can be an additive effect of inoculant and P fertiliser application on yield (Ronner et al., 2016). In our study, different nutrients in the applied fertiliser blend may have had contrasting impacts on the %Ndfa. While the N input from fertiliser may have suppressed N_2_-fixation (Salvagiotti et al., 2008), the additional P may have enhanced the %Ndfa (Pule-Meulenberg et al., 2011).

The total amount of N_2_ fixed was strongly affected by crop productivity components such as grain yield, biomass accumulation and plant height (Table 5). Unlike the %Nfda, total N_2_ fixation and soybean yields varied considerably between regions and years, probably due to different interacting production constraints (Fermont et al., 2009) such as soil characteristics, rainfall distribution, weed management, pest and disease incidence and time of sowing (Van Vugt et al., 2016). The combined application of fertiliser and inoculant increased the quantity of N_2_ fixed, biomass and grain yields (Table 4). This is in line with our findings that the amount of N_2_ fixed was associated with soil available P content (Table 5). Since soil exchangeable K content did not appear to be limiting (Table 1), the positive effect of K on N_2_-fixation (Table 5) may be due to a correlation between soil available P and exchangeable K (r = 0.35, p < 0.001). The effect of grain yield on total N_2_ fixation and vice versa (Table 5) suggests that adoption of yield improving crop management practices such as the correct sowing rate to achieve a good plant population (200,000–500,000 plants ha^−1^) and appropriate weed control (Table 5) will also result in larger quantities of N_2_ fixation. Therefore, our results suggest that farmers whose soybean crops are likely to fix large quantities of N_2_ are those who achieve good soybean yields, apply P fertiliser or have soils that are rich in available P, and adopt crop management practices that enhance biomass accumulation and grain yields. This is in line with several studies that have shown that including soybean in a maize-based system is a better investment if P fertiliser is applied to soybean (Ogoke et al., 2003, Kihara et al., 2010), since application of P fertiliser is known to enhance N accumulation by soybean (Jemo et al., 2006).

Soybean as a preceding crop improved maize grain yields, but this yield benefit was not affected by the soybean grain yields or biomass accumulation in 2010 (Table 9). Due to the limited number of plots included in the BNF trial in 2010, we also could not find correlations between the 2010 N_2_-fixation data and the rotational benefits of soybean to maize in 2011. A review of several studies in Sub-Saharan Africa shows that a cereal crop preceded by soybean takes up an additional 10–77 kg N ha^−1^ (Franke et al., 2017). This effect could be less in our study since the majority of farmers burnt or removed above-ground biomass from the field at harvest (Table 6), though there may have been a contribution of the below-ground biomass to the N economy (Wichern et al., 2008). The field N balance of soybean after grain removal is often negative (Vanlauwe and Giller, 2006, Salvagiotti et al., 2008, Mastrodomenico and Purcell, 2012) but it is usually still larger than in continuous maize without adequate N inputs (Peoples et al., 1995, Sanginga, 2003). In our study we did not measure N uptake by maize and the yield increase is likely to be a combination of N and non-N factors (Franke et al., 2017). Non-N rotational benefits could have included increased availability of P to maize following legumes (Carsky et al., 1997), suppression of root nematodes (Bagayoko et al., 2000) or other benefits (Franke et al., 2017). Non-N benefits may explain why yield increases of maize preceded by soybean were stronger in more productive fields where N was applied to maize (Table 8, Table 9).

### 4.3 Which farmers benefit most from N_2_-fixation and crop rotation?

For sustainable intensification to be acceptable to smallholder farmers, promoted technologies should be aligned to the local heterogeneous conditions and should result in immediate benefits for farmers (Vanlauwe et al., 2014). Surprisingly, gender strongly affected the %Ndfa, though this may be confounded with soil type since most participating female farmers were based in Dowa where soils were less sandy. Though interaction between soil texture and gender was not significant (p = 0.07), there is still a 93% likelihood that this was not by coincidence. Moreover, male farmers achieved better soybean grain yields (Table 5). This is in line with findings by Kilic et al. (2015) that female-managed plots in Malawi are 25% less productive than male-managed plots. Gender of the farmer was not related to any of the household socio-economic characteristics, which suggests that female farmers did not belong to poorer households than male farmers. Possibly, female farmers had less access to resources within the household, as was the case with climbing bean producers in Rwanda (Franke et al., 2016). Poor female farmers in Malawi are more likely to diversify into off-farm casual labour (*ganyu)* on wealthier farmers’ fields in exchange for basic food supplies (Bryceson, 2006, Simtowe, 2010). Time spent on off-farm activities may have negatively affected crop management and yields on female farmers’ fields. Men tend to allocate their time to high-value crops resulting in limited male labour inputs in female-managed fields (Kilic et al., 2015). Our results show that wealth of the household in terms of value of assets is positively associated with soybean grain yields (Table 5) and the absolute maize yield response to soybean (Table 9), probably because wealthier farmers have more fertile soils and can afford better management (Franke et al., 2014). These farmers may be in a better position to invest in nutrient inputs. The low value to cost ratio of fertiliser application to soybean and farmers’ perceptions that soybean does not require additional nutrients may hamper the adoption of inorganic fertiliser application in legumes (Kamanga et al., 2010, Van Vugt et al., 2016). However, our observation that the response to nutrient inputs to maize is enhanced by soybean as a previous crop implies that soybean cultivation can make fertiliser application to maize economically more attractive. Poor farmers who cannot afford improved varieties and fertiliser inputs may benefit less from including soybean in the crop rotation than wealthier farmers who can invest in improved inputs.

The results from both trials suggest that an improved integrated management including variety choice, external nutrient input application and other yield enhancing crop management practices, is associated with greater N_2_-fixation and residual effects of soybean on a subsequent maize crop. Thus, promoting the cultivation of soybean should be part of a wider Integrated Soil Fertility Management strategy (Vanlauwe et al., 2010). Farmers that have the means to invest in yield-enhancing technologies in both maize and soybean are likely to achieve the greatest benefits from incorporating soybean in maize-based rotations. Simply distributing soybean seed to support resource-poor smallholder farmers without further support is unlikely to be an effective development strategy.
